# A Telesurveillance System With Automatic Electrocardiogram Interpretation Based on Support Vector Machine and Rule-Based Processing

**DOI:** 10.2196/medinform.4397

**Published:** 2015-05-07

**Authors:** Te-Wei Ho, Chen-Wei Huang, Ching-Miao Lin, Feipei Lai, Jian-Jiun Ding, Yi-Lwun Ho, Chi-Sheng Hung

**Affiliations:** ^1^National Taiwan UniversityGraduate Institute of Biomedical Electronics and BioinformaticsTaipeiTaiwan; ^2^National Taiwan UniversityGraduate Institute of Communication EngineeringTaipeiTaiwan; ^3^National Taiwan UniversityDepartment of Electrical EngineeringTaipeiTaiwan; ^4^National Taiwan University HospitalTelehealth CenterTaipeiTaiwan

**Keywords:** telehealth care, telesurveillance system, electrocardiogram, ECG classification, support vector machine

## Abstract

**Background:**

Telehealth care is a global trend affecting clinical practice around the world. To mitigate the workload of health professionals and provide ubiquitous health care, a comprehensive surveillance system with value-added services based on information technologies must be established.

**Objective:**

We conducted this study to describe our proposed telesurveillance system designed for monitoring and classifying electrocardiogram (ECG) signals and to evaluate the performance of ECG classification.

**Methods:**

We established a telesurveillance system with an automatic ECG interpretation mechanism. The system included: (1) automatic ECG signal transmission via telecommunication, (2) ECG signal processing, including noise elimination, peak estimation, and feature extraction, (3) automatic ECG interpretation based on the support vector machine (SVM) classifier and rule-based processing, and (4) display of ECG signals and their analyzed results. We analyzed 213,420 ECG signals that were diagnosed by cardiologists as the gold standard to verify the classification performance.

**Results:**

In the clinical ECG database from the Telehealth Center of the National Taiwan University Hospital (NTUH), the experimental results showed that the ECG classifier yielded a specificity value of 96.66% for normal rhythm detection, a sensitivity value of 98.50% for disease recognition, and an accuracy value of 81.17% for noise detection. For the detection performance of specific diseases, the recognition model mainly generated sensitivity values of 92.70% for atrial fibrillation, 89.10% for pacemaker rhythm, 88.60% for atrial premature contraction, 72.98% for T-wave inversion, 62.21% for atrial flutter, and 62.57% for first-degree atrioventricular block.

**Conclusions:**

Through connected telehealth care devices, the telesurveillance system, and the automatic ECG interpretation system, this mechanism was intentionally designed for continuous decision-making support and is reliable enough to reduce the need for face-to-face diagnosis. With this value-added service, the system could widely assist physicians and other health professionals with decision making in clinical practice. The system will be very helpful for the patient who suffers from cardiac disease, but for whom it is inconvenient to go to the hospital very often.

## Introduction

Telehealth care is a global trend affecting clinical practice around the world. It allows for the remote care of patients at a distance using information and communication technology (ICT). Telehealth care is a continuous, automatic, real-time, and home-based remote monitoring system of patients that provides person-centered facilities to support individual health care. A previous study has reported that telehealth care may help patients and families to optimize adherence to therapy and may promote early intervention of abnormal signs by long-term telehealth monitoring [1]. In addition, several surveys in telehealth programs revealed beneficial results in clinical outcomes. A study of telemonitoring programs indicated that the all-cause mortality, the length of hospital stay, and the hospitalization rate were significantly reduced in telehealth users [2]. For these reasons, recent developments in Web-based telehealth care systems were designed to continually monitor the health status of chronic disease patients and elderly people [3-5]. People with heart disease problems, especially, should be warned to take particular care in daily life. However, it is difficult to follow up the situation of patients in real time and to provide early intervention in emergency cases. Fortunately, with the progress and development of telecommunication technologies, particularly in networks and electrical signal devices, telecom facilities have afforded telehealth care as an appropriate approach for disease management [6-9]. A real-time, computer-based support system is suitable for patients and health care providers in clinical practice [10]. Generally, information and communication technology has been recognized as an important tool in helping reduce health care costs while maintaining a high level of quality. A general-purpose telehealth care system must fully integrate remote management programs, including wireless telecommunication, a sensor network, a user interactive platform, and the information technology to deliver the synchronous service. Therefore, to provide ubiquitous monitoring and to offer value-added services, a comprehensive, reliable, and efficient data-reporting and analyzing system and its extendable modules must be established.

The electrocardiogram (ECG) is commonly used to detect abnormal heart rhythms and investigate the cause of heart abnormalities. An ECG, which can be acquired by a noninvasive procedure, is a transthoracic interpretation of the heart electrical activity over a period of time by using electrodes attached to the surface of the skin. In clinical practice, an ECG is a critical tool in diagnosing and identifying heart abnormalities by several features. Some features observed by ECGs are the RR interval (ie, the time measurement between the R waves of two heartbeats), the QRS complexes (ie, the duration of ventricular depolarization from Q wave to S wave), the ST segment (ie, the interval between ventricular depolarization and repolarization, between S wave and T wave), the T wave (ie, repolarization of the ventricles), and the amplitude of R-wave peaks (ie, electrical stimulus passing through the ventricular walls). An ECG also gives important information about human heart status related to critical healthy or unhealthy parameters. Most heart diseases can be detected by analyzing the ECG signal. The ECG is characterized by a cyclic occurrence of patterns with different frequency contents. A good ECG analysis method can accurately detect the morphological characteristics of the QRS complexes as well as the peaks. In the ECG analysis process, one of the most important procedures is to detect R-wave peaks. When the position of the R-wave peak is found, the locations of other feature points of ECG signals, such as Q peaks and S peaks, can be found by the relative position to the R-wave peak. Therefore, the accuracy of R-wave peak detection in ECG signals becomes very important. There have been several R-wave peak detection algorithms proposed in the past decades. Generally, these algorithms can be categorized into time-based detection algorithms [11-14], which are easy to implement but sometimes sensitive to noise, and frequency-based detection algorithms [15-18], which require more computation time but have better detection performance because of good robustness-to-interference, or noise, ratio.

In recent years, there were some research studies about atrial premature contraction (APC) heartbeat detection from ECG signals. Most algorithms of APC detection are time based and use the QRS morphology information for APC heartbeat classification [19-23]. On the other hand, some APC detection algorithms [24-26] are frequency based and adopt the Fourier transform or the wavelet transform. In these R-wave peak detection and APC detection algorithms, the support vector machine (SVM), the rule-based decision tree, the artificial neural network, or fuzzy logic are used as classifiers.

Over the past decades, many studies have put effort into ECG peak identification and heartbeat classification. However, few of them specifically focused on multidisease interpretation from ECG signals. Additionally, despite the numerous classification approaches in the literature, no study has convincingly demonstrated the hybrid model using a large, real-world ECG database.

In general, interpretation of ECG signals is a complicated and time-consuming task for cardiologists, especially when the data size is very large. Hence, to mitigate the increasing workload of cardiologists, and to provide continuous telehealth care and offer value-added service, the aim of this study was to construct a clinical decision support system (CDSS) with a knowledge-based ECG recognition program based on the support vector machine and rule-based processing approaches. The proposed software was designed to aid medical practitioners in decision making and clinical practice. The entire system included the automatic mechanism of data transmission, data storage, signal processing, and classification analysis. With the information from electronic medical records and analysis results, medical staff could use this telesystem to provide ubiquitous health care for patients.

## Methods

### Electrocardiogram Signal Analysis Using the Proposed Telesurveillance System

**Figure 1:**
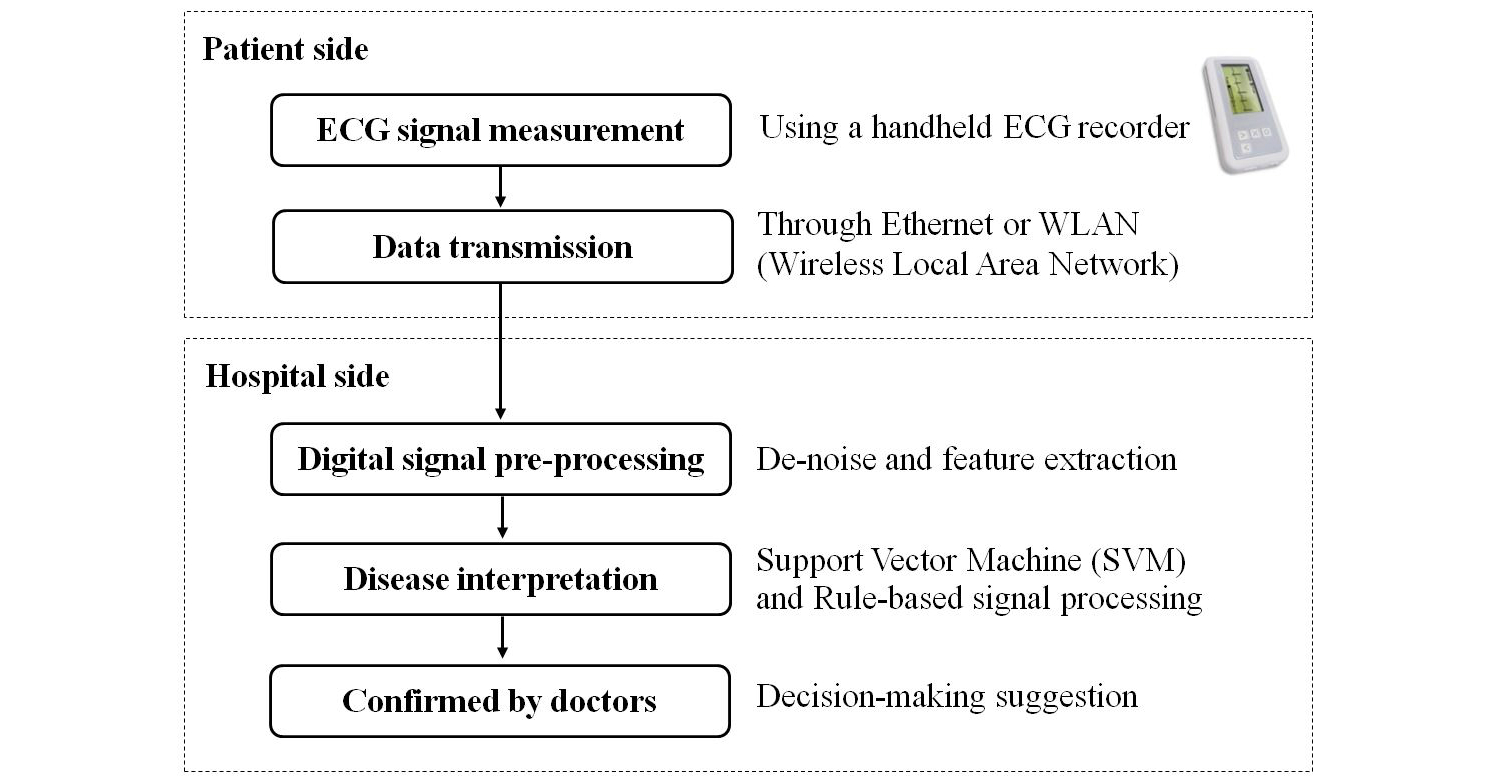
Flowchart of ECG signal analysis in the telesurveillance system. Patients use the handheld recorder to obtain the single-lead ECG signal, which will be automatically transmitted to the Telehealth Center at the NTUH for monitoring.

The data flow of ECG signal analysis is illustrated in Figure 1. In this study, we divided the flowchart into two parts. The first part represents the data flow on the patient side. The flowchart shows how we derived the ECG signal from patients. Patients can use the ECG recorder, which is similar in size to handheld mobile phones, to derive single-lead ECG signals as independently as possible. The recorder can securely and quickly transmit the measured data to the hospital server over Ethernet connection or the wireless local area network (WLAN). The other part of the flowchart shows the data processing on the hospital side. Data preprocessing is an important process for data analysis. We adopted the finite impulse response (FIR) filter to remove noise and the drift caused from the baseline. After noise reduction, we extracted the key features of the ECG waveforms and used SVM or rule-based processing to construct a classification model, which could suggest diagnoses. Finally, the medical practitioners were able to make decisions with the help of the suggested diagnoses from the system.

**Figure 2:**
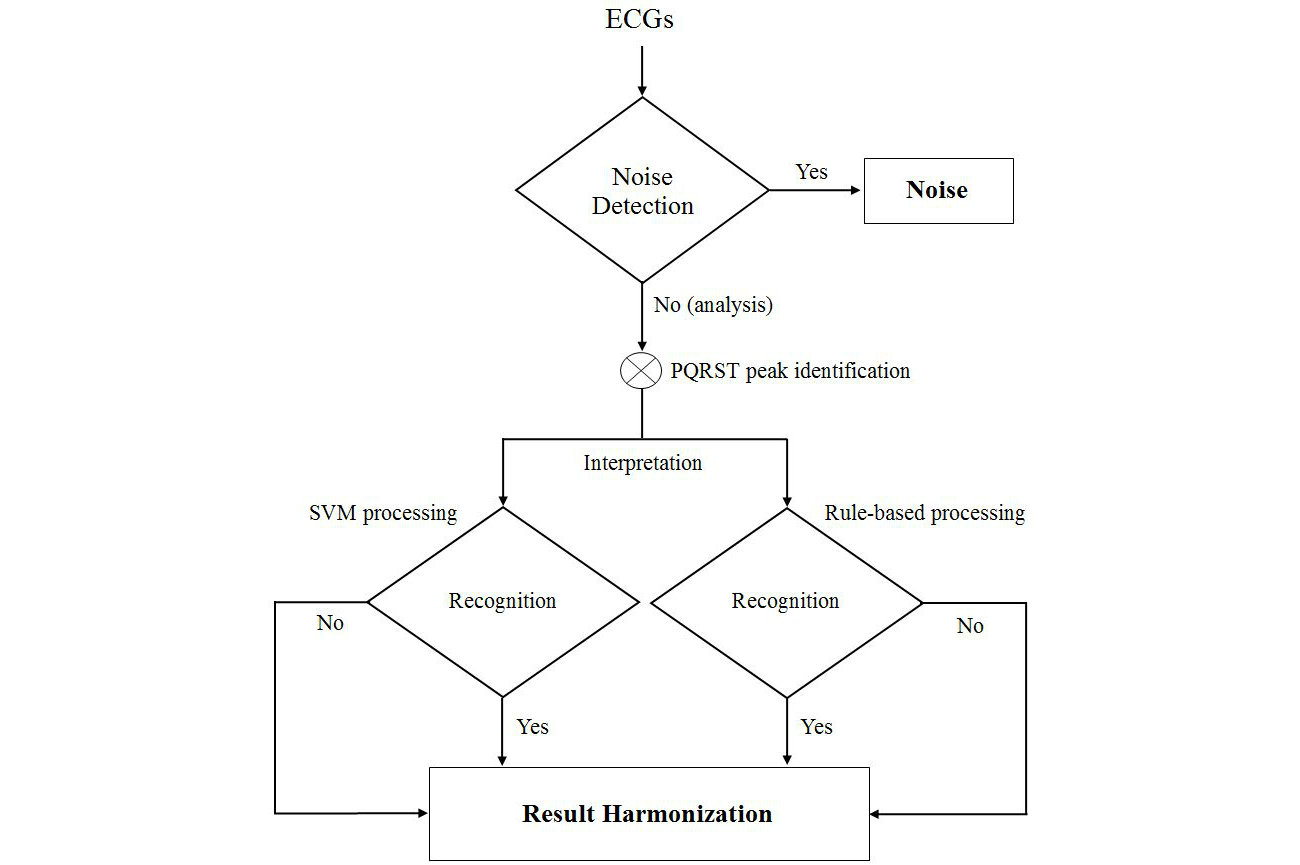
Flowchart of the automatic ECG recognition algorithm. Several preprocessing steps (ie, denoising, baseline removal, and feature extraction) and the classifiers of SVM and rule-based processing are applied to analyze the ECG signal.

The interpretation mechanism is the critical part of an automatic classification system. The process of the automatic ECG recognition algorithm is shown in Figure 2. We divided the process into four sections: noise reduction, peak estimation, feature extraction, and diagnosis interpretation. Noise reduction could enhance the signal part of the ECG from a contaminated record. Peak estimation was used to detect the locations of the P, Q, R, S, and T peaks for further analysis. Feature extraction was used to extract the key information of signals as the interpretation criteria of classifiers. Finally, we used the classifiers for the purpose of heartbeat status monitoring in this study.

**Figure 3:**
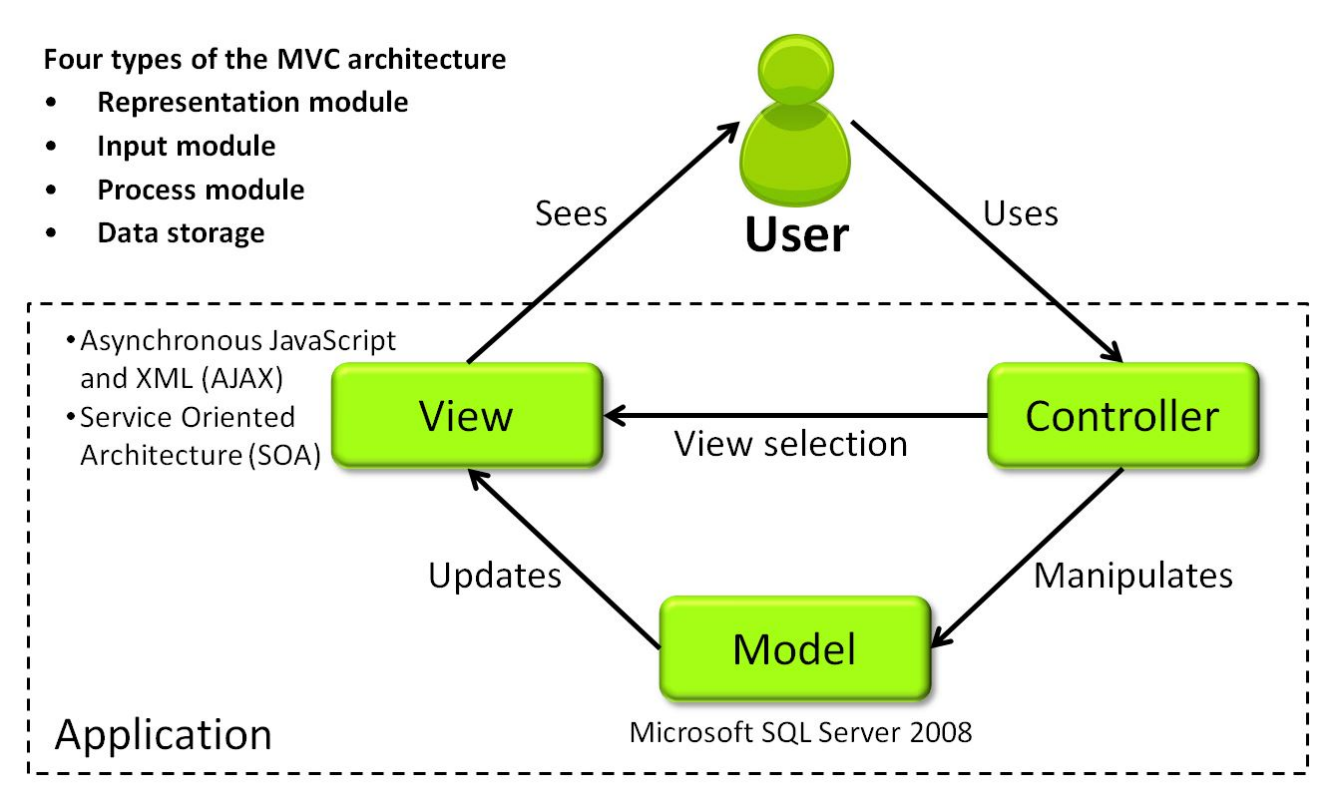
The high-level description of the user-environment system architecture, Model-View-Controller (MVC). Based on the MVC architecture, the modules of the platform can be clean, flexible, reusable, and extendable for programmers.

The clinical decision support system was implemented using the C# language in the ASP.NET Model-View-Controller (MVC) architecture. The model is an application object and the controller is a function between the user interface and input. The concept of MVC (see Figure 3) is to connect the human's mental model with the digital model, which exists in the computer. At the very least, the concept was adopted as a design pattern which is able to separate different sections. First, the user interface, including representation and the input control, is designed. Second, users can view and manage the data. Finally, the data bank will be updated. Microsoft Structured Query Language (SQL) Server 2008 was used for data computation and analysis. For the purpose of timely transmission and efficient delivery of the needed data to the user, the system was developed using the asynchronous JavaScript and XML (AJAX) technology and service-oriented architecture (SOA). AJAX, a group of client-side technologies, is based on existing standards that allows asynchronous communication by exchanging small amounts of data with the server in the background. The main purpose of AJAX is to enhance the speed, performance, and usability of Web applications. SOA is basically a collection of services that may be under the control of different ownership domains, and is able to interact, share, and exchange information without knowing the inner mechanism of the different systems. In this study, to provide individualized health management, we used the Web service to derive electronic medical records (EMRs) from the National Taiwan University Hospital (NTUH), which included such information as prescriptions, allergy records, laboratory data, and comorbidities.

Before analyzing ECG signals, the process of noise reduction was applied, as in Figure 2. Noise reduction was used to remove the interference and the baseline from signals. Its purpose is to address ECG enhancement and to accurately interpret a contaminated ECG signal. In this study, the denoising approach was based on a finite impulse response filter, which has become one of the most effective and popular denoising methods in many biomedical signal fields in recent years [27,28]. A band-pass FIR filter can reduce the noise and remove the baseline. The ECG signal has always suffered from the baseline drifting problem, which may lead to misdiagnosis if the drifting is severe. Therefore, baseline removal was very important to the ECG signal analysis. After removing the baseline, the locations and amplitudes of the P, Q, R, S, and T peaks can be determined accurately. Instead of using the median filter, which was adopted by many existing algorithms, we applied an innovative method to remove the baseline based on a gradient weighting function and a baseline ratio index [29]. These functions could improve the detection accuracy of the ECG R-wave peak for feature extraction, as discussed in the next section.

### Peak Estimation and Feature Extraction

The R-wave peak of an ECG complex signal is a dominant and essential characteristic which usually has the greatest height in a QRS complex. In this paper, R-wave peak candidates were identified by using the local maximum or minimum in a sliding window [30]. To enhance the detection accuracy, we took advantage of an adaptive peak-height thresholding method and a search-back method for sifting through R-wave peaks precisely. Moreover, the Q, S, P, and T peaks are also representative characteristic features. Their locations highly influence the accuracy of feature extraction. In the proposed system, several techniques were applied to estimate the P, Q, S, and T peaks accurately and efficiently. For efficiency, the sliding detection window technique and the second-order difference method were applied. For accuracy, the Mexican hat function was applied as a template-matching filter to approximate the PQRST complex. Based on the proposed algorithm, the Q, S, P, and T peaks can be detected in an accurate way even if the ECG signal suffers highly from noise.

The detection performance of R-wave peaks is based on the Massachusetts Institute of Technology-Beth Israel Hospital (MIT-BIH) arrhythmia database, which contains 48 half-hour, two-channel ambulatory ECG records. The characteristics of the MIT-BIH arrhythmia database include 11-bit resolution and a sampling frequency of 360 Hz. There are total 650,000 sampling points per ECG signal record. All 48 records, including 2546 atrial premature contraction heartbeats, 7130 ventricular premature contraction (VPC) heartbeats, and a total number of 109,494 heartbeats, were evaluated in the proposed method.

The features adopted in the classifiers of the entire system are summarized in Table 1. They were grouped into three parts. First, we employed a general extraction method based on the wavelet transform. It can extract both the detailed and the large-scale information. In this study, we applied three types of wavelet transforms: the spline 5/3 wavelet, the Cohen-Daubechies-Feauveau (CDF) 9/7 wavelet, and the Daubechies wavelet. Second, as another part of feature extraction, we calculated peak segments. For example, we first detected R-wave peaks, then the R-wave peak was utilized to calculate the vector between nearby peaks. Hence, we could derive all peak points of the ECG signal. Third, we acquired the features by computing the correlation and the segment lengths among these peak points. Most importantly, we established several features for specific diseases. For example, atrial fibrillation (AF) is the most common abnormal heart rhythm disease. The irregularity of RR intervals and the absence of P waves are used as the features to identify AF. Hence, we used the variant RR interval lengths to detect the irregular RR intervals and used fake P waves to detect the absence of P waves.

Furthermore, in a rule-based processing classifier, to detect the morphological characteristics of the ECGs we also applied the wave pattern and the time-based features among peaks, as seen in previous studies [31,32].

**Table 1:** Features of classifiers.

Model and extraction methods		Descriptions
**Support vector machine**		
	Wavelet transform 5/3, 9/7, and Daubechies	The maximum, minimum, mean, and variance using each wavelet transform. The number of features extracted by the three wavelet transforms is 12.
	Peak-segment features	Local maximums of RR interval widths/R-wave peak amplitudes in different scales Local minimums of RR interval widths/R-wave peak amplitudes in different scales Local means of RR interval widths/R-wave peak amplitudes in different scales Local variances of RR interval widths in different scales The number of local maximums between two R-wave peaks The rate of the case where the P peak does not exist Local mean of PR-segment lengths Local mean of QT-segment lengths Local mean of ST-segment lengths Local mean of P-wave widths Local mean of T-wave widths Local mean of QRS-complex width Local mean of P-wave amplitudes Local mean of T-wave amplitudes
**Rule based**		
	Amplitude and time analysis	The amplitude of the R-wave peak The amplitude of the S peak The amplitude ratio of the R-wave peak and the maximal amplitude of the S peak and the Q peak The distance between the Q and S peaks in a QRS complex The distance between the R and S peaks in a QRS complex The distance between the Q- and R-wave peaks in a QRS complex The ratio of the current RR interval to the local average RR interval

### Data Collection

The ECG data were collected from the telehealth program of the Telehealth Center at the National Taiwan University Hospital from February 14, 2012 to December 31, 2014. The dataset contained 213,420 ECGs from 530 patients. For classification, we divided the data into the training dataset and the validation dataset. We selected the data from 2012 as the training dataset, and the remaining data as the validation dataset. Out of 213,420 ECGs, the training dataset and validation dataset contained 26,181 (12.27%) and 187,239 (87.73%) ECGs, respectively. The training data were used to construct the SVM classification model, whereas the validation data were used to validate the accuracy of the models. The parameters of the ECG signals in this study were as follows: the time to acquire a continuous ECG signal was 15 seconds, the sampling frequency was 256 samples/second, the input dynamic range was ±2 mV, and the bandwidth was 0.004 Hz to 40 Hz.

### Diagnosis and Electrocardiogram Heartbeat Classification

For the purpose of ECG signal classifications, we applied the algorithm that is a combination of support vector machine and rule-based processing. The SVM [33] is a nonlinear classification method. It is a supervised learning model with automatic learning algorithms that analyze data patterns for classification and discrimination analysis. The concept of the SVM method is to transfer the input features into a multiple-dimensional space. In this space, a set of hyperplanes is constructed by the attributes transformed from the features. The ultimate goal of the SVM method is to generate the optimal hyperplanes that are used as the classification principles to separate all subjects [34]. The SVM method has become more and more popular in signal and image processing [35-37]. In our system, the radial basis function (RBF) kernel was applied for constructing SVM models, and the model parameter for the slack variable was set to 100.

The classification method of rule-based processing is to interpret ECGs using expert knowledge in designing. The method is generally suitable for analyzing the morphological characteristics of ECGs. For this reason, we could discriminate abnormal heartbeats, such as APC and VPC, using the QRS-wave pattern and the RR interval. Generally, these kinds of heartbeats do not have a normal morphology and impose an arrhythmic change in normal ECG patterns. Thus, we applied a rule-based, weighted Bayesian classifier to detect abnormal heartbeats. According to the medical definition of heartbeats, we applied the following rules for classification: (1) the current RR interval is smaller than the average RR interval, (2) the current QRS-complex width is larger than the average QRS-complex width, and (3) the amplitudes of the current R, S, and Q peaks are higher than those of other heartbeats.

Due to the diversity of the ECG waveforms and the purpose of optimization classification, we constructed an integrated surveillance decision algorithm that integrates the SVM model and rule-based processing according to their discrimination performance. In addition, the overfitting problem should be avoided as it may overrepresent the performance of models. Therefore, we did not discriminate the data using the principle “OR” among classifier results.

To test the performance of the proposed algorithm, the statistical indicators of sensitivity (SE), the positive prediction rate (+P), the detection error rate (DER), specificity (SP), and accuracy (ACC) were adopted for evaluating the results. An accurate algorithm will have higher SE, +P, SP, and ACC values and a smaller DER value. The formulas of SE, +P, and DER are listed in equations (1) and (2) where true positive (TP) is the number of the true cases that are successfully recognized as true cases, false negative (FN) is the number of true cases that are regarded as false cases, false positive (FP) is the number of false cases that are treated as true cases, and true negative (TN) is the number of false cases that are validly identified as false cases.

SE(%) = TP/(TP+FN),+P(%) = TP/(TP+FP), DER(%) = (FP+FN)/(TP+FN) (1)

SP(%) = TN/(TN+FP), ACC(%) = (TP+TN)/(TP+TN+FP+FN) (2)

To validate the capability of the proposed classification models, we conducted a retrospective study using the confirmation ECG data that were used for diagnosis by cardiologists as the gold standard to verify the models.

## Results

### Telesurveillance System

**Figure 4:**
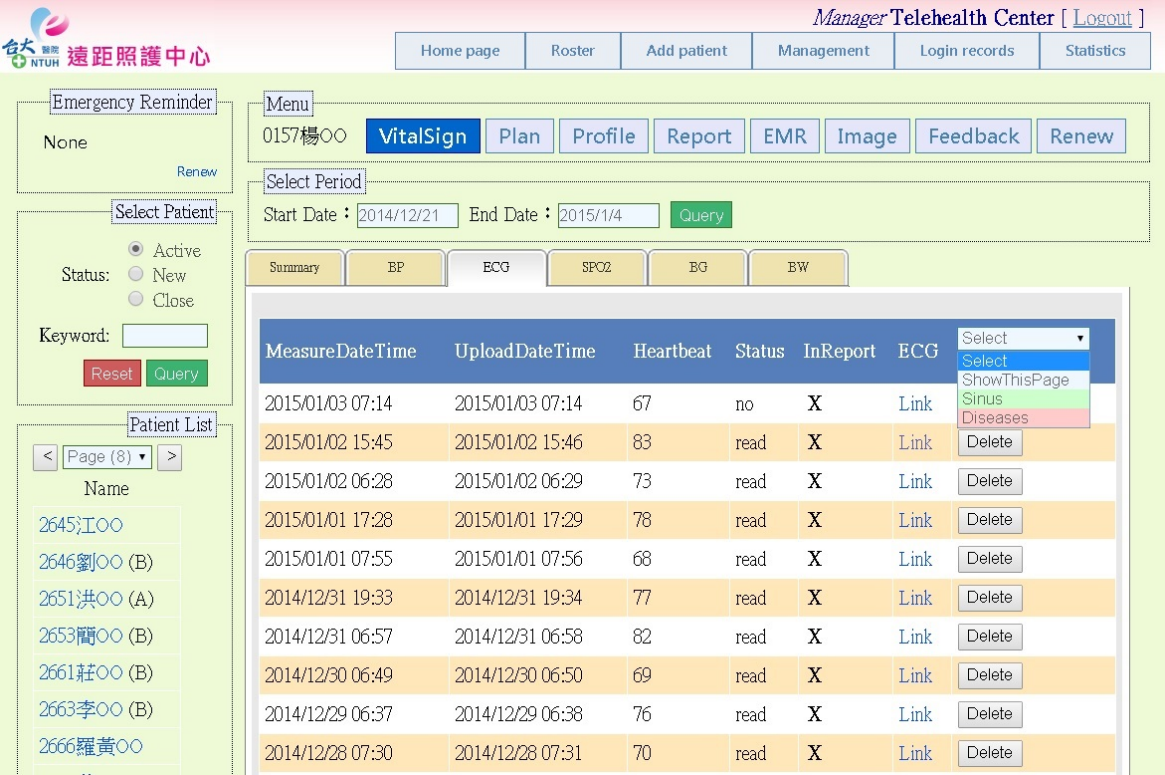
A screenshot of the telesurveillance system. Users are able to access the required information on the platform, such as patients’ biometric data, electronic medical records, and monthly statistical reports.

To provide ubiquitous telehealth care, a telesurveillance system at the Telehealth Center of the NTUH was deployed under an SOA framework with the Health Level Seven (HL7) standard. By providing long-term informative interaction and long-term health monitoring, the presented telehealth care system is more than a health monitoring system—it is also helpful for clinical decision making. The service provided must be able to take care of routines and subroutines and act as a health information center to share the data among heterogeneous platforms, such as hospital information systems and health information systems. Hence, our system successfully provides a continuous, real-time, secure, Web-based telehealth care service for both patients and medical staff. A screenshot of our system is shown in Figure 4. The menu on the left side of the screen includes a patient list with the personal serial number and name of each patient. The right-hand section contains the following menu items: (1) VitalSign, which contains uploaded biometric data, including single-lead ECGs, blood pressure, heart rate, oximetry, and glucometry—in diabetic patients with impaired fasting glucose and impaired glucose tolerance, (2) Plan, which contains telephone interview records and health care planning, (3) Profile, which contains the patient’s individual profile, (4) Report, which is the monthly statistical report, (5) EMR, which is the electronic medical record, including the history of prescriptions, medications, allergies, and laboratory data at the NTUH, (6) Image, which contains uploaded wound photos, (7) Feedback, which contains the user’s satisfaction survey and nutritional assessment, and (8) Renew, which refreshes the page and data. For example, the section VitalSign illustrates the main uploaded information, including the recording date/time, uploading date/time, and estimated heartbeat. It also provides the list of patients’ ECGs. Users are able to access the required information by clicking the tabs. Moreover, in order to reduce the workload of medical practitioners, the user can switch between sinus and disease ECGs. These tags are labeled by the automatic classification mechanism. Sinus data are normal rhythm ECGs and disease data are ECGs associated with any disease. After selection, the ECG data will be displayed on the platform.

**Figure 5:**
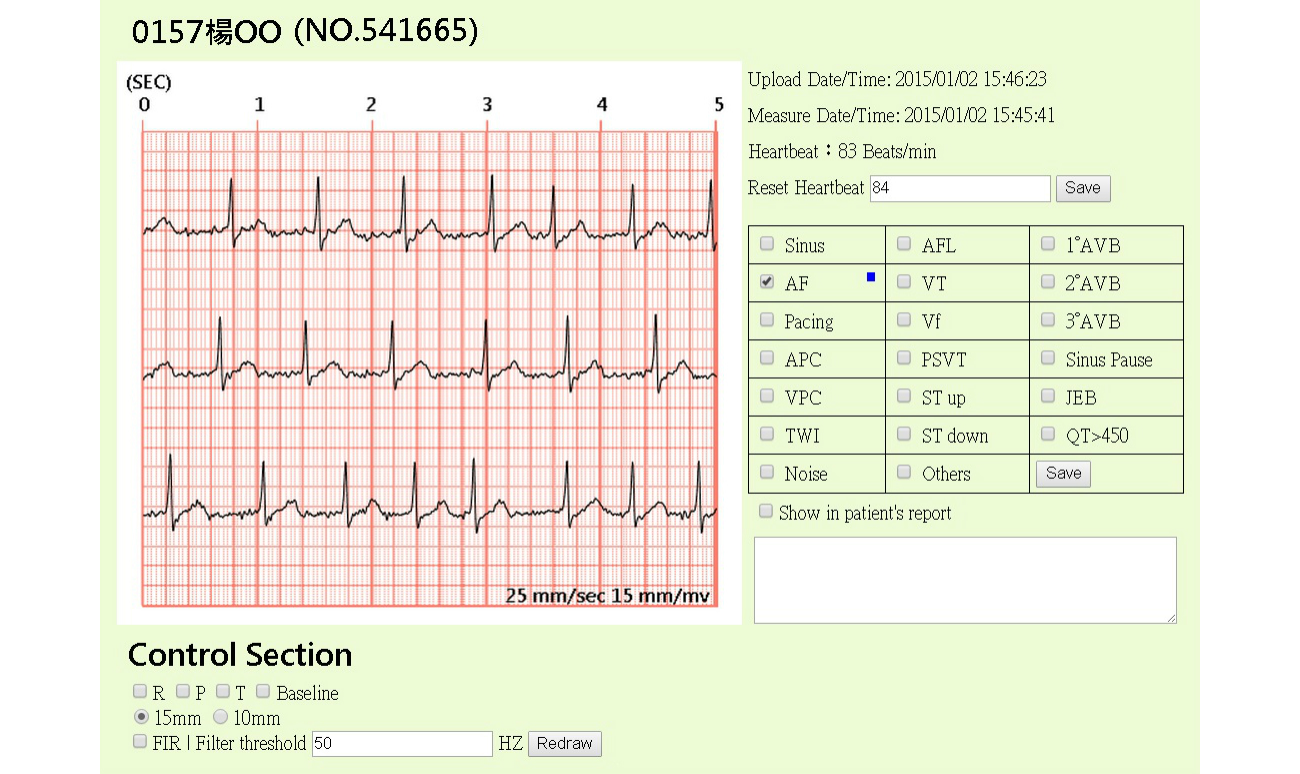
A screenshot of ECG diagnosis using the telesurveillance system. The ECG waveform and the corresponding classification suggestions are revealed on the screen. The suggested heartbeat classification is marked with a blue dot. Health professionals can make decisions using this information in clinical practice.

To provide value-added service, the system is equipped with an automatic interpretation function to help medical personnel in clinical practice. We designed a Web-based user interface for medical staff, which can review the ECG data on the platform and make a decision with corresponding classification suggestions. The diagnostic interface of an ECG record is illustrated in Figure 5. The left-hand section shows a continuous 15-second ECG signal with the common standard unit. In a standard ECG, the width of a single, small square represents 0.2 seconds and the height of the square is 0.5 mV. Moreover, users can click on the bottom, left-hand buttons to indicate the R, P, and T peaks, and the baseline of the ECG. By the same token, they can not only switch the height of the ECG figure to 15 mm/mV or to 10 mm/mV, but they can also use the filter to eliminate the frequency noise. Items on the right-hand side include the date/time of uploading and measuring, estimated heartbeats, diagnosis selection, and the marked area. If “Show in patient’s report” is selected, the ECG and judgment will appear in the patient’s monthly statistical report. At the Telehealth Center of the NTUH, there are 20 types of ECG diagnoses built into the database, such as sinus rhythm, atrial fibrillation, and first-degree atrioventricular block (AVB1). We employ an icon to easily represent the diagnosis suggestion by classification models. The information is relayed to the cardiologist who makes the final clinical decision and health care suggestions. For example, in Figure 5, the “AF” cell is labeled with a blue dot by the model in this ECG case. Hence, the physician could pay more attention to this icon, whether the suggestion is consistent with their diagnosis or not. In particular, the supported information is very important with some complicated data—it could provide assistance to physicians in enhancing the accuracy of decision making. After diagnosis, for high quality of care, the serious abnormal data would immediately alert case managers, who could then make phone calls to patients or their caregivers.

### Peak Evaluation Results

The performance of the proposed R-wave peak detection algorithm, based on a total of 48 records from the MIT-BIH arrhythmia database, was analyzed. Out of a total number of 109,494 heartbeats in the MIT-BIH arrhythmia database, the algorithm detected 73 false negatives and 134 false positives, giving it a detection error rate of 0.19%. The sensitivity of the algorithm was 99.93% (109,371 true positives/[109,371 true positives plus 73 false negatives]). The algorithm had a positive prediction rate of 99.88% (109,371 true positives/[109,371 true positives plus 134 false positives]) (see Table 2). In particular, for healthy and semihealthy cases, the average detection error rate was 0.1% (SD 0.002). One thing to notice is that the R-wave peak detection algorithm was very simple to implement without using any transform-domain methods, such as the Fourier transform and the wavelet transform. This algorithm also used the adaptive threshold method to increase the ECG R-wave peak detection accuracy rate and reduce the numbers of false positives and false negatives by considering the cases of irregularity and noise-like peaks on ECG signals. When implemented by MATLAB, the average detection time for each 30-minute ECG dataset in the MIT-BIH database was less than 0.65 seconds.

**Table 2:** Performance of R-wave peak detection algorithm.

Characteristics of dataset and algorithm		n or %
**Type of beats, n**		
	Total beats	109,494
	True positives	109,371
	False negatives	73
	False positives	134
**Algorithm performance measure, %**		
	Detection error rate	0.19
	Positive prediction rate	99.88
	Sensitivity	99.93

### Descriptive Statistics

The automatic ECG classification mechanism proposed by this study was evaluated using the diagnostic data at the Telehealth Center of the NTUH. The distribution of the ECG data is shown in Table 3. There were 213,420 heartbeats from 530 patients measured between February 14, 2012 and December 31, 2014. Overall, the number of sinus, disease, and noise cases from the entire dataset of 213,420 heartbeats were 151,040 (70.77%), 54,218 (25.40%), and 10,514 (4.93%), respectively. Additionally, the heartbeat problem that occurred most often was atrial fibrillation (21,580/213,420, 10.11%). Other common problems—sorted by the number of cases—out of 213,420 heartbeats were atrial premature contraction (11,181, 5.24%), atrial flutter (7858, 3.68%), first-degree atrioventricular block (6304, 2.95%), and pacemaker rhythm (6040, 2.83%). To compare the differences between the training dataset and the validation dataset, the proportion of the sinus, disease, and noise cases in the training dataset was made to be similar to that of the validation dataset.

**Table 3:** Electrocardiogram dataset from 530 patients from the Telehealth Center of the National Taiwan University Hospital.

Diagnosis		Number of heartbeats, n (%)		
		Total (n=213,420)	Training dataset (n=26,181)	Validation dataset (n=187,239)
All		213,420 (100)	26,181 (100)	187,239 (100)
Sinus		151,040 (70.77)	18,429 (70.39)	132,611 (70.82)
Uncertain		8162 (3.82)	1593 (6.08)	6569 (3.51)
**Disease** ^a^				
	All^b^	54,218 (25.40)	6159 (23.52)	48,059 (25.67)
	AF	21,580 (10.11)	2232 (8.53)	19,348 (10.33)
	AFL	7858 (3.68)	800 (3.06)	7058 (3.77)
	Pacemaker rhythm	6040 (2.83)	1433 (5.47)	4607 (2.46)
	APC	11,181 (5.24)	1234 (4.71)	9947 (5.31)
	VPC	732 (0.34)	107 (0.41)	625 (0.33)
	TWI	3064 (1.44)	307 (1.17)	2757 (1.47)
	ST-segment down	1156 (0.54)	92 (0.35)	1064 (0.57)
	AVB1	6304 (2.95)	341 (1.30)	5963 (3.18)
	JEB	263 (0.12)	39 (0.15)	224 (0.12)
	QT>450	5 (0)	0 (0)	5 (0)
Noise		10,514 (4.93)	1904 (7.27)	8610 (4.60)

^a^Atrial fibrillation (AF), atrial flutter (AFL), atrial premature contraction (APC), ventricular premature contraction (VPC), T-wave inversion (TWI), first-degree atrioventricular block (AVB1), junctional escape beat (JEB), QT-segment length is more than 450 milliseconds (QT>450).

^b^Since two or more problems may occur at a heartbeat at the same time, the sum of individual disease heartbeats is more than the number of all disease heartbeats combined.

### Automatic Electrocardiogram Classification Results

In this study, we used the validation data to obtain an objective performance evaluation with several indicators. The capability of the proposed ECG classification mechanism is shown in Table 4. The experimental results show that the accuracy, sensitivity, and specificity in sinus (ie, normal rhythms) cases were 53.32% ([47,036 true positive plus 52,804 true negative]/187,239 total), 35.47% (47,036 true positive/[47,036 true positive plus 85,575 false negative]), and 96.67% (52,804 true negative/[1824 false positive plus 52,804 true negative]), respectively.

Since we hope that, when the disease case occurs, the clinician can be informed, it is important to prevent the classification model from missing any possible disease data. Therefore, the model with higher specificity for sinus cases and higher sensitivity for disease cases is preferred. Table 4 shows that, in the disease case, our model yielded a sensitivity of 98.50% (47,339 true positive/[47,339 true positive plus 720 false negative]). In the sinus case, the model yielded a specificity of 96.67%.

For the detection performances of specific diseases, the recognition models generated sensitivity values of 92.70% (17,935 true positive/[17,935 true positive plus 1413 false negative]) in atrial fibrillation, 89.10% (4105 true positive/[4105 true positive plus 502 false negative]) in pacemaker rhythm, 88.60% (8813 true positive/[8813 true positive plus 1134 false negative]) in atrial premature contraction, 72.98% (2012 true positive/[2012 true positive plus 745 false negative]) in T-wave inversion, 62.21% (4391 true positive/[4391 true positive plus 2667 false negative]) in atrial flutter, and 62.57% (3731 true positive/[3731 true positive plus 2232 false negative]) in first-degree atrioventricular block. Moreover, the accuracy, sensitivity, and specificity to detect the noise cases were 81.17% ([6984 true positive plus 144,995 true negative]/187,239 total), 81.11% (6984 true positive/[6984 true positive plus 1626 false negative]), and 81.17% (144,995 true negative/[33,634 false positive plus 144,995 true negative]), respectively. Since the noisy ECG signals could be identified by the algorithm accurately, it could be adjusted by denoising approaches to yield good-quality ECG signals.

**Table 4:** Electrocardiogram classification performance for the dataset from the Telehealth Center of the National Taiwan University Hospital.

Diagnosis		Characteristics of dataset and algorithm						
		Type of beats, n				Algorithm performance measure, %		
		True positive	False negative	False positive	True negative	Accuracy	Sensitivity	Specificity
Sinus		47,036	85,575	1824	52,804	53.32	35.47	96.66
**Disease** ^a^								
	All	47,339	720	94,842	44,338	48.96	98.50	31.86
	AF	17,935	1413	20,357	147,534	88.37	92.70	87.88
	AFL	4391	2667	12,530	167,651	91.88	62.21	93.05
	Pacemaker rhythm	4105	502	117,876	64,756	36.78	89.10	35.46
	APC	8813	1134	48,838	128,454	73.31	88.60	72.45
	VPC	317	308	4595	182,019	97.38	50.72	97.54
	TWI	2012	745	22,623	161,859	87.52	72.98	87.74
	ST-segment down	471	593	10,007	176,168	94.34	44.27	94.63
	AVB1	3731	2232	15,771	165,505	90.39	62.57	91.30
	JEB	30	194	4698	182,317	97.39	13.39	97.49
	QT>450	1	4	10,630	176,604	94.32	20.00	94.32
Noise		6984	1626	33,634	144,995	81.17	81.12	81.17

^a^Atrial fibrillation (AF), atrial flutter (AFL), atrial premature contraction (APC), ventricular premature contraction (VPC), T-wave inversion (TWI), first-degree atrioventricular block (AVB1), junctional escape beat (JEB), QT-segment length is more than 450 milliseconds (QT>450).

## Discussion

### Principal Findings

In this study, a telesurveillance system with automatic recognition of the ECG in real time was implemented. Our system was intentionally designed for monitoring and classifying the ECG signals of telehealth users who are being cared for at home. Ultimately, ECGs could not only be transmitted to the hospital over the telecommunication system, but could also be recognized using automatic ECG classifiers for offering a suggestion for diagnosis. Therefore, the system provides the 24-hour service every day. It can automatically identify abnormal ECGs and send alarms to health care providers. In ECG preprocessing, we used a denoising approach based on an FIR filter and performed baseline drift removal with a gradient weighting function. Both techniques can enhance the signal portion of a contaminated ECG record and improve the accuracy of feature extraction. Next, a fixed sliding window, an adaptive peak-height thresholding scheme, and a search-back method were applied for ECG peak detection. According to the preliminary results of R-wave peak evaluation from the MIT-BIH arrhythmia database, our algorithm achieved a detection error rate of 0.19%, a sensitivity of 99.93%, and a positive prediction rate of 99.88%. Moreover, wavelet transforms, relative locations, matched filters, and the regularity test were also employed for feature extraction. For abnormal heartbeat classification, we adopted the interpretation approaches of the support vector machine and rule-based processing. The experimental results of the proposed ECG classification mechanism showed the classifiers yielded a specificity of 96.66% for normal heartbeats, a sensitivity of 98.50% for disease cases, and an accuracy of 81.17% for noise cases. For diagnosing specific heartbeat problems, the interpretation model generated sensitivities of 92.70% for atrial fibrillation, 89.10% for pacemaker rhythm, 88.60% for atrial premature contraction, 72.98% for T-wave inversion, 62.21% for atrial flutter, and 62.57% for first-degree atrioventricular block. For medical staff, they would be able to upload the ECG signals of patients through this clinical decision support system. Then, the immediately automatic interpretation of the ECG could provide physicians with a suggested diagnosis to help them make a decision accurately. This system is very helpful especially when the data size is very large. Moreover, we also integrated electronic medical records into the system, which include such information as prescriptions, food allergies, and drug allergies. With this information, the medical staff could provide more adequate advice to patients.

### Limitations

There were some limitations to this study. First, the SVM model is not suitable to use with the imbalanced data, since it tends to classify the instances into the majority class. To overcome this problem, we first applied the rule-based approach to recognize the minority class. The rule-based classifier could immediately detect the disease cases using some specific features. Second, we adopted a genetic algorithm to generate the most relevant features for constructing SVM models, whereas the total features were selected as input features for training in order to create optimal classifiers. As well, additional rule-based features were required to augment the current automated classification models to consider all of the features for classifying. For the rule-based classifier, all selected features were determined after discussions with ECG-domain knowledge experts (ie, hospital doctors), and also from in-depth consultation of several ECG textbooks. Third, we classified abnormal heartbeats with specific features. Hence, for these classifiers it is hard to identify heartbeat problems without significant features. For example, ventricular tachycardia (VT) and ventricular fibrillation (VF) usually do not have normal waves, complexes, and segments due to improper electrical activity and the uncoordinated contraction of the cardiac muscle. Moreover, the number of cases of these diseases is fairly small and it is not suitable to construct SVM models. These kinds of ECGs may usually be classified into the noise class. Fortunately, with the progress and development of the implantable cardioverter defibrillator (ICD), this therapy could save patients with sudden cardiac disease. Finally, the accuracy of ECG diagnosis depends on the coding of cardiologists [38,39]. This is an innate disadvantage of big database analysis. Nevertheless, these kinds of studies reveal real-world information that can be used for medical science research studies, and they offer a meaningful contribution in the form of generating evidence to solve current medical issues. Besides, this study resulted in 96.18% (205,258/213,420) readable ECGs—the ECGs that were not classified as uncertain cases in Table 3. We believe that the reliability of this data is sufficient for conducting research studies and for making diagnosis suggestions for physicians.

### Comparison With Prior Work

With the advances in modern telecommunication technologies, telehealth care is one of the trends in medical treatment. Previous studies have confirmed that telehealth care is an efficient approach in disease management [2,40-42]. The telehealth care system in this study is not only a health monitoring system, but also a tool that assists in decision making. Fortunately, our previous studies have shown that the Telehealth Center of the National Taiwan University Hospital has provided effective telehealth care for chronic cardiovascular disease patients and has reduced medical costs and the burden on caregivers [43-45]. A previous study has also indicated that the data analytics in the telehealth care system could assist clinicians at the point of care [46]. In this study, we established an automatic mechanism for ECG signal collection, transmission, and processing, and then used this massive amount of data to implement a clinical decision support system, which was codesigned by the clinicians at the NTUH.

ECG R-wave peak detection is one of the most important parts of a fully automated ECG analysis algorithm. Many R-wave peak detection algorithms have been proposed. The methods in some previous studies [13,15,47] are time-domain based, and those in two other studies [48,49] are transform-domain based. Cui [13] proposed an algorithm based on zero-crossing counting. It achieved a sensitivity of 99.8% and a detection error rate of 0.6%. The algorithm by Chen et al [15] mainly applied morphology and background noise removal, and achieved a sensitivity of 99.7% and a detection error rate of 0.7%. Wang et al [47] proposed another QRS-detection algorithm—it generated a sensitivity of 99.8% and a detection error rate of 0.5%. Arzeno et al [48] proposed an algorithm that is based on the discrete wavelet transform (DWT) with a sigma-delta modulator—it achieved a sensitivity of 98.0% and a detection error rate of 2.8%. The algorithm by Hamilton and Tompkins [49] used the biorthogonal spline wavelet and applied the Mallat algorithm to detect feature points—it had a sensitivity of 99.7% and a detection error rate of 0.5%. By contrast, the real-time R-wave peak detection algorithm adopted in our system used slopes to find the local maxima or minima within a fixed time slot. The QRS R-wave peak usually happened at the local maxima or minima with the largest change of slope. In addition, an adaptive thresholding scheme, the regularity of heartbeats, the matched filter, and the sharpness of the peak were also adopted for R-wave peak detection. Evaluation results showed that the proposed method achieved a positive prediction rate of 99.88%, a sensitivity of 99.93%, and a detection error rate of 0.19% when applied to data from the MIT-BIH arrhythmia database, which indicates better performance than that of the other methods. Moreover, since R-wave peak candidate sifting is applied, our algorithm can be implemented in an efficient way.

In recent years, there were several related research studies about detecting cardiac anomalies from ECG signals. The first study proposed an arrhythmia disease diagnosis method based on the artificial neural network (ANN) classifier using the University of California at Irvine (UCI) 12-lead arrhythmia data. Their model classified ECGs into normal or abnormal (ie, arrhythmia) cases. They obtained a sensitivity and a specificity of 93.8% and 93.1%, respectively [50]. Another method that applied the feed-forward artificial neural network to identify normal, VPC, and other heartbeats was proposed by Ince et al [51]. For the MIT-BIH arrhythmia database, Ince et al’s method achieved 99.4% sensitivity and 98.9% specificity for identifying normal heartbeats, 93.4% sensitivity and 93.3% specificity for determining VPC heartbeats, and 87.5% sensitivity and 97.8% specificity for other heartbeats. Sankari and Adeli [52] proposed a mobile cardiac monitoring system for identifying three cardiac pathologies: atrial fibrillation, atrioventricular block, and myocardial infarction. The system yielded a sensitivity of 95.0%—detecting 95.0% of the pathologies—and a specificity of 100%. However, the system was tested using 60 simulated pathologic ECG datasets rather than a big database. A recent study that investigated the autoregressive model for atrial fibrillation screening was proposed by Parvaresh and Ayatollahi [53]. The experimental results using the MIT-BIH AF database showed that the model’s sensitivity and specificity were 96.1% and 93.2%, respectively [53]. Similarly, in another research study, Lian et al [54] developed an AF detector based on the change of RR intervals. It yielded 94.3% sensitivity and 95.1% specificity when applied to the MIT-BIH atrial fibrillation database, and 98.1% sensitivity and 77.0% specificity when applied to the MIT-BIH arrhythmia database. However, it only generated a specificity of 84.1% for non-AF detection when applied to the MIT-BIH normal sinus rhythm database. In fact, we also tested the performance of our algorithm using the MIT-BIH database. Most of the VPC heartbeats were detected successfully with an average sensitivity value of 98.08% and an average specificity value of 99.31%. For APC feature extraction and classification, the proposed algorithm yielded an average sensitivity value of 97.45% and an average specificity value of 99.52%. Compared with the previous studies, our methods have an even better performance when applied to the MIT-BIH database.

Although these studies have algorithms that achieve good performance for ECG classification, they are generally not suitable for multiple heartbeat problem diagnoses. In addition, the performance of these models was evaluated using the MIT-BIH database rather than real-world data, which can be significantly affected by various environmental factors and can be much more complicated to analyze.

To make the proposed telesurveillance system really helpful to practical clinics, we developed an automatic ECG interpretation algorithm using real-world, multiple-diagnosed ECG data from the telehealth care program. The proposed system yielded a much higher specificity for normal cases and a much higher sensitivity for disease cases than those of other algorithms. As a result, our mechanism is reliable enough to obviate the need for the physician’s diagnosis and confirmation.

### Conclusions

Via the telesurveillance system, the telehealth care and communication devices, and the automatic ECG interpretation mechanism, telehealth users can be monitored and cared for at home anytime, whereby real-time ECG signals are collected, transmitted, and displayed, and the corresponding classification suggestions are revealed on the system. Furthermore, this paper presents several methods for ECG signal preprocessing and classification. Traditional techniques aim at identifying heartbeats and adjusting the waveforms of ECG signals. In contrast, our proposed interpretation mechanism combines SVM and rule-based processing, and is intentionally designed to automatically analyze the ECG signals of patients in the telehealth care service system. With this value-added service, this intelligent system could widely assist physicians and other health professionals with decision-making tasks in clinical practice, which is important for making users accept remote medical assistance technologies in general.

## Abbreviations

+P: positive prediction rate
ACC: accuracy
AF: atrial fibrillation
AFL: atrial flutter
AJAX: asynchronous JavaScript and XML
ANN: artificial neural network
APC: atrial premature contraction
AVB1: first-degree atrioventricular block
CDF: Cohen-Daubechies-Feauveau
CDSS: clinical decision support system
DER: detection error rate
DWT: discrete wavelet transform
ECG: electrocardiogram
EMR: electronic medical record
FIR: finite impulse response
FN: false negative
FP: false positive
HL7: Health Level Seven
ICD: implantable cardioverter defibrillator
ICT: information and communication technology
JEB: junctional escape beat
MIT-BIH: Massachusetts Institute of Technology-Beth Israel Hospital
MVC: Model-View-Controller
NTUH: National Taiwan University Hospital
QT>450: QT-segment length is more than 450 milliseconds
RBF: radial basis function
SE: sensitivity
SP: specificity
SOA: service-oriented architecture
SQL: Structured Query Language
SVM: support vector machine
TN: true negative
TP: true positive
TWI: T-wave inversion
UCI: University of California at Irvine
VF: ventricular fibrillation
VPC: ventricular premature contraction
VT: ventricular tachycardia
WLAN: wireless local area network

## Multimedia Appendix 1

CONSORT-EHEALTH checklist V1.6.2 [55].
