# Supplementary material for: A Telesurveillance System With Automatic Electrocardiogram Interpretation Based on Support Vector Machine and Rule-Based Processing
Source: JMIR Med Inform. 2015 May 7;3(2):e21. doi: 10.2196/medinform.4397 (PMC4440896; doi:10.2196/medinform.4397)
Supplement: Supplementary file 1 [file medinform_v3i2e21_app1.pdf]

CONSORT-EHEALTH Checklist V1.6.2 Report

(based on CONSORT-EHEALTH V1.6), available at [http://tinyurl.com/consort-ehealth-v1-6].

4397

Date completed

4/1/2015 3:14:04

by

Jian-Jiun Ding

A Tele-surveillance System with Automatic ECG Interpretation based on Support Vector Machine and Rule-based Processing

TITLE

1a-i) Identify the mode of delivery in the title

The title of the manuscript is:

A "Tele-surveillance System" with Automatic ECG Interpretation based on Support Vector Machine and Rule-based Processing'

It contains the term "tele-surveillance system", which is related to remote healthcare and communication.

1a-ii) Non-web-based components or important co-interventions in title

Automatic ECG Interpretation based on Support Vector Machine and Rule-based Processing

1a-iii) Primary condition or target group in the title

There is no primary condition or target group in the title.

ABSTRACT

1b-i) Key features/functionalities/components of the intervention and comparator in the METHODS section of the ABSTRACT

Methods: We established a tele-surveillance system with an automatic ECG interpretation mechanism. The system included: (1) automatic ECG signal transmission via telecommunication; (2) ECG signal processing, including noise elimination, peak estimation, and feature extraction; (3) automatic ECG interpretation based on the Support Vector Machine classifier and rule-based processing; (4) displaying ECG signals and their analyzing results.

1b-ii) Level of human involvement in the METHODS section of the ABSTRACT

We analyzed 213,420 ECG signals that were diagnosed by cardiologists as the gold standard to verify the classification performance.

1b-iii) Open vs. closed, web-based (self-assessment) vs. face-to-face assessments in the METHODS section of the ABSTRACT

We analyzed 213,420 ECG signals that were diagnosed by cardiologists as the gold standard to verify the classification performance.

1b-iv) RESULTS section in abstract must contain use data

In the clinical ECG database from the Telehealthcare Center of NTUH, the experimental results showed that the ECG classifier yielded a specificity value of 96.7% for normal rhythm detection, a sensitivity value of 98.5% for disease recognition.

1b-v) CONCLUSIONS/DISCUSSION in abstract for negative trials

There is no discussion about negative trials.

INTRODUCTION

2a-i) Problem and the type of system/solution

Telehealthcare is a global trend affecting the clinical practice around the world. It offers the remote care of patients at a distance using the information and communication technology (ICT). Telehealthcare is a continuous, automatic, real-time, and home-based remote monitoring system of patients by providing person-centered facilities to support for individual healthcare.

:

:

Although over the past decades, many studies put effort to ECG peak identification and heartbeat classification. However, few of them specifically focused on multi-disease interpretation from ECG signals. Additionally, despite the numerous classification approaches in the literature, no study has convincingly demonstrated the hybrid model using the real large ECG database.

2a-ii) Scientific background, rationale: What is known about the (type of) system

A previous study has reported that telehealthcare may help patients and families to optimize adherence to therapy and promote early intervention of abnormal signs by long-term telehealth monitoring [1]. Besides, several surveys in telehealth programs revealed beneficial results in clinical outcomes. A study of telemonitoring programs indicated that the all-cause mortality, the length of hospital stay, and the hospitalization rate were significantly reduced in telehealth users [2]. For these reasons, recent developments in web-based telehealthcare systems were designed to continually monitor the health status of chronic disease patients and the elderly people [3-5]. Especially, people with heart disease problems should be warned to take particular care in daily life. However, it is hard to real-time follow up the situation of patients and to provide the early intervention in emergency cases. Fortunately, with the progress and development of telecommunication technologies, particularly in networks and electrical signal devices, telecom facilities have afforded telehealthcare an appropriate approach for disease management [6–9]. A real time computer-based support system is suitable for patients and healthcare providers in clinical practice [10]. Generally, the information and communication technology has been recognized as an important direction to reduce the healthcare cost while maintaining a high level of quality. A general-purpose telehealthcare system must fully integrate remote management programs, including wireless telecommunication, sensor network, a user interactive platform, and the information technology to deliver the synchronous service. Therefore, to provide ubiquitous monitoring and to offer value-added services, a comprehensive, reliable, and efficient data reporting and analyzing system and its extendable modules must be established.

METHODS

3a) CONSORT: Description of trial design (such as parallel, factorial) including allocation ratio

In general, interpretation the ECG signal is a complicated and time-consuming task for cardiologists, especially when the data size is very large. Hence, to mitigate the loading of cardiologists, provide a continuous telehealthcare, and offer the value-added service, the aim of this study is to construct a clinical decision support system (CDSS) with the knowledge-based ECG recognition program based on the support vector machine (SVM) and rule-based processing approaches. The proposed software was designed to aid medical practitioners in decision-making and clinical practice. The entire system included the automatic mechanism of data transmission, data storage, signal processing, and classification analysis. With the information of electronic medical records and analysis results, the medical staffs could use this tele-system to provide ubiquitous healthcare for patients.

3b) CONSORT: Important changes to methods after trial commencement (such as eligibility criteria), with reasons

The manuscript is not related to trial improvement. It is about heartbeat classification.

3b-i) Bug fixes, Downtimes, Content Changes

The manuscript is not related to bug fixes, downtimes, and content changes.

4a) CONSORT: Eligibility criteria for participants

The ECG data were collected from the telehealth program of the Telehealth Center in the National Taiwan University Hospital (NTUH) from 2012/2/14 to 2014/12/31. There were 213,420 ECGs from 530 patients.

4a-i) Computer / Internet literacy

The proposed tele-surveillance system can be used even if the user is not familiar with the internet or computer.

4a-ii) Open vs. closed, web-based vs. face-to-face assessments:

The proposed tele-surveillance system is only applied for ECG signal analysis. It is not used for trial. One just use the system to analyze the status of heartbeat. If the abnormal case happens, the patient should be trialed face-by-face.

4a-iii) Information giving during recruitment

There were 213,420 ECGs from 530 patients. All of them have signed consent forms.

4b) CONSORT: Settings and locations where the data were collected

The training and test ECG data were collected from the telehealth program of the Telehealth Center in the National Taiwan University Hospital (NTUH) from 2012/2/14 to 2014/12/31.

4b-i) Report if outcomes were (self-)assessed through online questionnaires

The web-based trial was not applied and there was no online questionnaires.

4b-ii) Report how institutional affiliations are displayed

the Telehealth Center in the National Taiwan University Hospital (NTUH), Taipei, Taiwan

5) CONSORT: Describe the interventions for each group with sufficient details to allow replication, including how and when they were actually administered

5-i) Mention names, credential, affiliations of the developers, sponsors, and owners

National Taiwan University (grant no. NTU-CESRP-104R7608-3)

5-ii) Describe the history/development process

The history / development process was not included in the text.

5-iii) Revisions and updating

The system is not the revision or the updating version of the previous one.

5-iv) Quality assurance methods

The quality assurance method was not discussed.

5-v) Ensure replicability by publishing the source code, and/or providing screenshots/screen-capture video, and/or providing flowcharts of the algorithms used

Figs. 1, 2, and 3 are the flowchart of the proposed system.
Figs. 4 and 5 are the screenshots of the tele-surveillance system

5-vi) Digital preservation

There is no digital preservation.

5-vii) Access

The tele-surveillance system has no limitation for users.

5-viii) Mode of delivery, features/functionalities/components of the intervention and comparator, and the theoretical framework

The interpretation mechanism is the critical part of an automatic classification system. The process of the auto ECG recognition algorithm is shown in Figure 2. We divided the process into four sections: noise reduction, peak estimation, feature extraction, and diagnosis interpretation. Noise reduction could enhance the signal part of the ECG from a contaminated record. Peak estimation is to detect the locations of P, Q, R, S, and T peaks for further analysis. Feature extraction is to extract the key information of signals as the interpretation criteria of classifiers. Finally, we used the classifiers for the purpose of heartbeat status monitoring in this study.
The clinical decision support system was implemented using the C# language in the ASP.Net Model-View-Controller (MVC) architecture. The model is an application object and the controller is a function between the user interface and input. The concept of MVC (Figure 3) is to connect human's mental model and the digital model exists in the computer. At least, the concept is adopted as a design pattern which is able to separate different sections. First, the user interface including representation and the input control is designed. Then, users could view and manage the data. Finally, the data bank will be updated. Microsoft SQL Server 2008 was used for data computation and analysis. For the purpose of timely transmission and efficiently pushing the needed data to the user, the system was developed using the Asynchronous JavaScript and XML (AJAX) and Service Oriented Architecture (SOA). AJAX, a group of client-side technologies, is based on existing standards that allows asynchronous communication by exchanging small amounts of data with the server in the background. The main purpose of AJAX is to enhance the speed, performance and usability of web applications. SOA is basically a collection of services that may be under the control of different ownership domains, and is able to interact, share, and exchange information without knowing the inner mechanism. In this study, to provide individualized health management, we used the web service to derive Electronic Medical Records (EMR) of the NTUH, such as prescriptions, allergy records, laboratory data, and comorbidities.

Before analyzing ECG signals, the process of noise reduction is applied, as in Figure 2. It is to remove the interference and the baseline from signals. Its purpose is to address ECG enhancement and to accurately interpret a contaminated ECG signal. In this study, the denoising approach is based on a Finite Impulse Response (FIR) filter, which has become one of the effective and popular denoise methods in many biomedical signal fields in recent years [27,28]. A bandpass FIR filter can reduce the noise and remove the baseline. Because the ECG signal is always suffered from the baseline drifting problem which may lead to misdiagnose if the drifting is severe. Therefore, baseline removal is very important to the ECG signal analysis. After removing the baseline, the locations and amplitudes of P, Q, R, S, and T peaks can be determined accurately. Instead of using the median filter, which was adopted by many existing algorithms, we applied an innovative method to remove the baseline based on a gradient weighting function and a baseline ratio index [29]. These functions could improve the detection accuracy of the ECG R-wave peak for feature extraction in the next section.

Peak Estimation and Feature Extraction
The R-wave peak of an ECG complex signal is a dominant and essential characteristic which usually has the highest height in a QRS complex. In this paper, R-wave peak candidates were identified by using the local maximum or minimum in a sliding window [30]. To enhance the detection accuracy, we take advantage of an adaptive peak-height thresholding method and a search-back method for sifting R-wave peaks precisely. Moreover, the Q, S, P, and T peaks are also representative characteristic features. Their locations highly influence the accuracy of feature extraction. In the proposed system, several techniques are applied to estimate P, Q, S, and T peaks accurately and efficiently. For efficiency, the sliding detection window technique and the second-order difference method are applied. For accuracy, the Mexican hat function is applied as a template matching filter to approximate the PQRST complex. Based on the proposed algorithm, the Q, S, P, and T peaks can be detected in an accurate way even if the ECG signal is highly suffered from noise.
The detection performance of R-wave peaks based on the Massachusetts Institute of Technology (MIT)-Boston's Beth Israel Hospital (BIH) arrhythmia database, which contains 48 half-hour two-channel ambulatory ECG records. The characteristics of the MIT-BIH arrhythmia database are 11-bit resolution and have the sampling frequency of 360Hz. There are total 650,000 sampling points per ECG signal record. All forty-eight records, including 2,546 Atrial Premature Contraction (APC) heartbeats, 7,130 Ventricular Premature Contraction (VPC) heartbeats, and a total number of 109,494 heartbeats, are evaluated in the proposed method.
The features adopted in the classifiers of the entire system are summarized in Table 1. They were grouped into three parts. First, we employed a general extraction method based on the wavelet transform. It can extract both the detail and the large-scale information. In this research, we applied three types of wavelet transforms: the spline 5/3 wavelet, the Cohen-Daubechies-Feauveau (CDF) 9/7 wavelet, and the Daubechies wavelet. Another part of feature extraction is to calculate peak-segments. For example, firstly, we detect R-wave peaks. Then, the R-wave peak is utilized to calculate the vector between nearby peaks. Hence, we could derive all peak points of the ECG signal. Finally, we acquire the features by computing the correlation and the segment lengths among these peak points. Most importantly, we establish several features for specific diseases. For example, the Atrial Fibrillation (AF) is the most common abnormal heart rhythm disease. The irregularity of RR intervals and the absence of P waves are used as the features to identify AF. Hence, we use the variance RR interval lengths to detect the irregular RR intervals and use the fake P waves to detect the absence of P waves. Furthermore, in a rule-based processing classifier, to detect the morphological characteristics of the ECGs, we also applied the wave pattern and the time-based features among peaks, as the work in [31,32].

5-ix) Describe use parameters
The parameters of the ECG signals in this study were as follows: the time to acquire a continuous ECG signal is 15 seconds, the sampling frequency is 256 samples/second, the input dynamic range is ±2 millivolts, and the bandwidth is 0.004 ~ 40Hz.

5-x) Clarify the level of human involvement

The ECG training and test data were acquired from 530 cardiac disease patients.

5-xi) Report any prompts/reminders used

There is no prompts/reminders used.

5-xii) Describe any co-interventions (incl. training/support)

There is no intervention.

6a) CONSORT: Completely defined pre-specified primary and secondary outcome measures, including how and when they were assessed
In this study, we used the validation data to obtain an objective performance evaluation with several indicators. The capability of the proposed ECG classification mechanism is shown in Table 4. The experimental results show that the accuracy, sensitivity, and specificity in sinus (normal rhythms) cases were 53.3% [(47,036 plus 52,804) out of 187,239], 35.5% [47,036 out of (47,036 plus 85,575)], and 96.7% [52,804 out of (1,824 plus 52,804)], respectively.
Since we hope that, when the disease case occurs, the clinician can be informed, it is important to prevent the classification model from missing any possible disease data. Therefore, the model with higher specificity for sinus cases and higher sensitivity for disease cases is preferred. Table 4 shows that, in the disease case, our model yielded the sensitivity of 98.5% [47,339 out of (47,339 plus 720)]. In the sinus case, the model yielded the sensitivity of 96.7%.

For the detection performances of specific diseases, the recognition models generated the sensitivity values of 92.7% [17,935 out of (17,935 plus 1,413)] in Atrial Fibrillation, 89.1% [4,105 out of (4,105 plus 502)] in Pacemaker rhythm, 88.6% [8,813 out of (8,813 plus 1,134)] in Atrial Premature Contraction, 73.0% [2,012 out of (2,012 plus 745)] in T-wave Inversion, 62.2% [4,391 out of (4,391 plus 2,667)] in Atrial Flutter, and 62.6% [3,731 out of (3,731 plus 2,232)] in First-degree Atrio-Ventricular Block, respectively. Moreover, the accuracy, sensitivity, and specificity to detect the noise case are 81.2% [(6,984 plus 144,995) out of 187,239], 81.1% [6,984 out of (6,984 plus 1,626)], and 81.2% [144,995 out of (33,634 plus 144,995)], respectively. Since the noisy ECG signal could be identified by the algorithm accurately, it could be adjusted by denoising approaches to yield a good quality ECG signal.
6a-i) Online questionnaires: describe if they were validated for online use and apply CHERRIES items to describe how the questionnaires were designed/deployed

There is no online questionnaire.

6a-ii) Describe whether and how “use” (including intensity of use/dosage) was defined/measured/monitored
The proposed software was designed to aid medical practitioners in decision-making and clinical practice. The entire system included the automatic mechanism of data transmission, data storage, signal processing, and classification analysis. With the information of electronic medical records and analysis results, the medical staffs could use this tele-system to provide ubiquitous healthcare for patients.

6a-iii) Describe whether, how, and when qualitative feedback from participants was obtained
There is no description about qualitative feedback.
6b) CONSORT: Any changes to trial outcomes after the trial commenced, with reasons
There is no change of the trial outcome.
7a) CONSORT: How sample size was determined
7a-i) Describe whether and how expected attrition was taken into account when calculating the sample size
There were 213,420 ECGs from 530 patients.
7b) CONSORT: When applicable, explanation of any interim analyses and stopping guidelines
There is no interim analysis and stopping guideline.
8a) CONSORT: Method used to generate the random allocation sequence
We selected the data acquired in 2012 as the training dataset, and the remaining data (acquired in 2013 and 2014) as the validation dataset. The training dataset and validation dataset were 26,181 (12.3%) and 187,239 (87.7%), respectively.
8b) CONSORT: Type of randomisation; details of any restriction (such as blocking and block size)
We assigned the data as a training or a test one according to when it was acquired.
9) CONSORT: Mechanism used to implement the random allocation sequence (such as sequentially numbered containers), describing any steps taken to conceal the sequence until interventions were assigned
We assigned the data as a training or a test one according to when it was acquired.
10) CONSORT: Who generated the random allocation sequence, who enrolled participants, and who assigned participants to interventions
The sequence was acquired from the doctors in National Taiwan University Hospital (NTUH).
11a) CONSORT: Blinding - If done, who was blinded after assignment to interventions (for example, participants, care providers, those assessing outcomes) and how
11a-i) Specify who was blinded, and who wasn't
The system is not related to trial.
11a-ii) Discuss e.g., whether participants knew which intervention was the "intervention of interest" and which one was the "comparator"
This was not described.
11b) CONSORT: If relevant, description of the similarity of interventions
There is no similarity of interventions.
12a) CONSORT: Statistical methods used to compare groups for primary and secondary outcomes
To test the performance of the proposed algorithm, the statistical indicators of sensitivity (SE), the positive prediction rate (+P), the detection error rate (DER), specificity (SP), and accuracy (ACC) are adopted for evaluating the results. An accurate algorithm will have higher SE, +P, SP, ACC values and a smaller DER value. The formulas of SE, +P, and DER are listed in (1) and (2) where TP (true positive) is the number of the true cases that are successfully recognized as true cases, FN (false negative) is the number of true cases that are regarded as false cases, FP (false positive) is the number of false cases that are treated as true cases, and TN (true negative) is the number of false cases that are validly identified as false cases.

To validate the capability of proposed classification models, we conducted a retrospective study using the confirmation ECGs data that were diagnosed by cardiologists as the gold standard to verify the models.
12a-i) Imputation techniques to deal with attrition / missing values
In our simulations, there is no attrition / missing value problem.
12b) CONSORT: Methods for additional analyses, such as subgroup analyses and adjusted analyses
There is no subgroup analyses and adjusted analyses.

RESULTS

13a) CONSORT: For each group, the numbers of participants who were randomly assigned, received intended treatment, and were analysed for the primary outcome
The participants were not separated into groups.
13b) CONSORT: For each group, losses and exclusions after randomisation, together with reasons
The participants were not separated into groups.
13b-i) Attrition diagram
The manuscript is not about usage, dose, and engagement
14a) CONSORT: Dates defining the periods of recruitment and follow-up
There is no recruitment and follow-up.
14a-i) Indicate if critical "secular events" fell into the study period
There is no secular event.
14b) CONSORT: Why the trial ended or was stopped (early)
The trial was not stopped early. It was ended when the data is enough.
15) CONSORT: A table showing baseline demographic and clinical characteristics for each group
This work is about an ECG analysis telehealth system. There is no clinical characteristics for each group.
15-i) Report demographics associated with digital divide issues
We have not addressed it. We will include the information in the final version if necessary.
16a) CONSORT: For each group, number of participants (denominator) included in each analysis and whether the analysis was by original assigned groups
16-i) Report multiple "denominators" and provide definitions
The denominator has been given. For example,

The performance of the proposed R-wave peak detection algorithm based on total 48 records from the MIT-BIH arrhythmia database obtains a detection error rate of 0.2% [(73 plus 134) out of 109,494], a sensitivity of 99.9% [109,371 out of (109,371 plus 73)], and a positive prediction of 99.9% [109,371 out of (109,371 plus 134)] (Table 2).
16-ii) Primary analysis should be intent-to-treat

This work is about an ECG analysis telehealth system, not about treatment. So intent-to-treat is not applicable. Instead, we apply the sensitivity, the accuracy, and the specificity to evaluate the performance.

In this study, we used the validation data to obtain an objective performance evaluation with several indicators. The capability of the proposed ECG classification mechanism is shown in Table 4. The experimental results show that the accuracy, sensitivity, and specificity in sinus (normal rhythms) cases were 53.3% [(47,036 plus 52,804) out of 187,239], 35.5% [47,036 out of (47,036 plus 85,575)], and 96.7% [52,804 out of (1,824 plus 52,804)], respectively.
Since we hope that, when the disease case occurs, the clinician can be informed, it is important to prevent the classification model from missing any possible disease data. Therefore, the model with higher specificity for sinus cases and higher sensitivity for disease cases is preferred. Table 4 shows that, in the disease case, our model yielded the sensitivity of 98.5% [47,339 out of (47,339 plus 720)]. In the sinus case, the model yielded the sensitivity of 96.7%.

For the detection performances of specific diseases, the recognition models generated the sensitivity values of 92.7% [17,935 out of (17,935 plus 1,413)] in Atrial Fibrillation, 89.1% [4,105 out of (4,105 plus 502)] in Pacemaker rhythm, 88.6% [8,813 out of (8,813 plus 1,134)] in Atrial Premature Contraction, 73.0% [2,012 out of (2,012 plus 745)] in T-wave Inversion, 62.2% [4,391 out of (4,391 plus 2,667)] in Atrial Flutter, and 62.6% [3,731 out of (3,731 plus 2,232)] in First-degree Atrio-Ventricular Block, respectively. Moreover, the accuracy, sensitivity, and specificity to detect the noise case are 81.2% [(6,984 plus 144,995) out of 187,239], 81.1% [6,984 out of (6,984 plus 1,626)], and 81.2% [144,995 out of (33,634 plus 144,995)], respectively. Since the noisy ECG signal could be identified by the algorithm accurately, it could be adjusted by denoising approaches to yield a good quality ECG signal.

17a) CONSORT: For each primary and secondary outcome, results for each group, and the estimated effect size and its precision (such as 95% confidence interval)

This work is about an ECG analysis telehealth system, not about treatment. So the confidence interval is not included. Instead, we apply the sensitivity, the accuracy, and the specificity to evaluate the performance.

17a-i) Presentation of process outcomes such as metrics of use and intensity of use

The manuscript is not about the use of dose.

17b) CONSORT: For binary outcomes, presentation of both absolute and relative effect sizes is recommended

There is no binary outcome.

18) CONSORT: Results of any other analyses performed, including subgroup analyses and adjusted analyses, distinguishing pre-specified from exploratory

There is no any other analyses performed.

18-i) Subgroup analysis of comparing only users

There is no subgroup analysis.

19) CONSORT: All important harms or unintended effects in each group

ECG analysis is secure and there is no harm or unintended effects in each group.

19-i) Include privacy breaches, technical problems

There is no privacy breach and technical problem in our work.

19-ii) Include qualitative feedback from participants or observations from staff/researchers

There is no qualitative feedback from participants or observations from staff/researchers.

DISCUSSION

20) CONSORT: Trial limitations, addressing sources of potential bias, imprecision, multiplicity of analyses

20-i) Typical limitations in ehealth trials

There were some limitations to this study. First, the SVM is not suitable to the imbalanced data since it tends to classify the instances to the majority class. To overcome this problem, we applied the rule-based approach to recognize the minority class. The rule-based classifier could immediately detect the disease cases using some specific features. Second, we adopted a genetic algorithm to generate the most relevant features for constructing SVM models, whereas the total features were selected as input features for training in order to create optimal classifiers. Besides, additional rule-based features were required to augment the current automated classification models to consider all of the features for classifying. For the rule-based classifier, all selected features are determined after we discuss with ECG-domain knowledge experts (hospital doctors) and also have a deep study from several ECG textbooks. Third, we classify abnormal heartbeats with features; hence, for these classifiers, it is hard to identify the heartbeat problem without significant features. For example, the ventricular tachycardia (VT) and the ventricular fibrillation (VF) usually do not have normal waves, complexes, and segments due to the improper electrical activity and the uncoordinated contraction of the cardiac muscle. Moreover, the number of cases of these diseases is fairly small and it is not suitable to construct SVM models. These kinds of ECGs may usually be classified to the noise class. Fortunately, with the progress and the development of the implantable cardioverter defibrillator (ICD), the therapy could save the patients with the sudden cardiac disease. Finally, the accuracy of ECG diagnosis depends on the coding of cardiologists [38,39]. This is an innate disadvantage of big database analysis. Nevertheless, these kinds of studies reveal the real-world information for medical science researches and offer a meaningful contribution of generating an evidence to solve the present medical issues. Besides, the readable ECGs of this study are near to 96.2% (205258/213420). We believe that the reliability of data is sufficient to conduct researches and make suggestions for physicians.

21) CONSORT: Generalisability (external validity, applicability) of the trial findings

21-i) Generalizability to other populations

No generalisability of the trial findings was addressed.

21-ii) Discuss if there were elements in the RCT that would be different in a routine application setting

There was no element in the RCT.

22) CONSORT: Interpretation consistent with results, balancing benefits and harms, and considering other relevant evidence

22-i) Restate study questions and summarize the answers suggested by the data, starting with primary outcomes and process outcomes (use)

Via the tele-surveillance system, the telehealthcare and communication devices, , and the automatic ECG interpretation mechanism, telehealth users can be monitored and taken care at home anytime, whereby real-time ECG signals are collected, transmitted, displayed, and the corresponding classification suggestions are revealed on the system. Furthermore, this paper presents several methods for ECG signal pre-processing and classification. Unlike the traditional techniques that aim at identifying heartbeats and adjusting the waveforms of ECG signals, our proposed interpretation mechanism that combines the SVM and rule-based processing is intentionally designed for automatically analyzing the ECG signals of patients in the telehealthcare service system. With this value-added service, the intelligent system could widely assist physicians and other health professionals with the decision-making tasks in clinical practice, which is important for making users accept remote medical assistance technologies in general.

22-ii) Highlight unanswered new questions, suggest future research

No unanswered new question was addressed.

Other information

23) CONSORT: Registration number and name of trial registry

We will include it in the final version if it is necessary.

24) CONSORT: Where the full trial protocol can be accessed, if available

We will include it in the final version if it is necessary.

25) CONSORT: Sources of funding and other support (such as supply of drugs), role of funders
National Taiwan University (grant no. NTU-CESRP-104R7608-3)
X26-i) Comment on ethics committee approval
The collected ECG data has been approved by the IRB. We will attach the form in the final version if necessary.
x26-ii) Outline informed consent procedures
The consent was obtained offline.
X26-iii) Safety and security procedures
There is no risk for ECG data collection
X27-i) State the relation of the study team towards the system being evaluated
The authors declare that they have no conflict of interest
